# Association between CD14 Promoter -159C/T Polymorphism and the Risk of Sepsis and Mortality: A Systematic Review and Meta-Analysis

**DOI:** 10.1371/journal.pone.0071237

**Published:** 2013-08-19

**Authors:** An-qiang Zhang, Cai-li Yue, Wei Gu, Juan Du, Hai-yan Wang, Jianxin Jiang

**Affiliations:** State Key Laboratory of Trauma, Burns and Combined Injury, Institute of Surgery Research, Daping Hospital, Third Military Medical University, Chongqing, China; Yale School of Public Health, United States of America

## Abstract

**Background:**

Recent studies on the association between CD14-159C/T polymorphism and sepsis showed inconclusive results. Accordingly, we conducted a comprehensive literature search and a meta-analysis to determine whether the CD14-159C/T polymorphism conferred susceptibility to sepsis or was associated with increased risk of death from sepsis.

**Methodology:**

Data were collected from the following electronic databases: PubMed, Embase, Medline, Web of Knowledge, and HuGE Navigator, with the last report up to June 15, 2012. The odds ratio (OR) and 95% confidence interval (CI) were used to assess the strength of association. We summarized the data on the association between CD14-159C/T polymorphism and sepsis in the overall population and subgroup by ethnicity and sepsis subtype.

**Principal Findings:**

A total of 16 studies on sepsis morbidity (1369 cases and 2382 controls) and 4 studies on sepsis mortality (731 sepsis patients) met the inclusion criteria for meta-analysis. Overall analysis showed no strong evidences of association with sepsis susceptibility under any genetic model. However, slight associations were found in Asian populations (dominant model: OR = 1.38, 95%CI = 0.96–1.98, P = 0.08) and septic shock patients (dominant model: OR = 1.72, 95%CI 1.05–2.83, P = 0.03; allelic model: OR = 1.52, 95%CI 1.09–2.12, P = 0.01) in the stratified analysis. Moreover, there was borderline association between CD14-159C/T and sepsis mortality under the dominant genetic model (OR = 1.44, 95%CI = 0.98–2.11, P = 0.06).

**Conclusions/Significance:**

This meta-analysis suggests that the CD14-159C/T polymorphism may not be a significant susceptibility factor in the risk of sepsis and mortality. Only weak associations were observed in Asian populations and septic shock patients. More studies based on larger sample sizes and homogeneous sepsis patients are needed to confirm these findings.

## Introduction

Sepsis is regarded as a condition of systemic inappropriate inflammation response of the host organism to the invasion of microorganisms, which is the leading cause of death in critically ill patients. Although pathogen invasion is important determinant of sepsis, it is difficult to identify prognostic factors that may predict susceptibility and mortality in sepsis. Classic population genetic techniques suggest a strong genetic component to both the risk of developing sepsis and the subsequent outcome in terms of survival. A study of adoptees, for example, indicated a substantial inherited increase in the risk of premature death from infection [1]. Large number of publications aim at association of genetic polymorphism and sepsis [2]. These polymorphisms mainly include variants in genes coding for proteins involved in the recognition of bacterial pathogens and response to bacterial pathogens. Cluster of differentiation 14 (CD14) has been studied extensively.

CD14 serves as a central pattern recognition molecule in innate immunity, and the binding of LPS-CD14-MD2 complex to TLR4 could activate NF-κB signaling pathway, and initiate an inflammatory response [3]. CD14 is expressed on the surface of monocytes, macrophages, and neutrophils as membrane-bound CD14 and in the serum as soluble CD14 and its expression may be partially regulated at the genetic level [4]. Increased membrane-bound or soluble CD14 levels are well-established biological risk factors for the development of sepsis and subsequent vital organ dysfunction both in animal experiments [5] and in critically ill patients [6]–[8]. CD14-deficient mice demonstrated a strong resistance to infection [9]. Taken together, these results suggest that CD14 may play a pivotal role in the pathogenesis of sepsis.

The human CD14 gene is located on chromosome 5q31.1. Many single nucleotide polymorphisms (SNPs) have been identified in the CD14 gene (http://www.ncbi.nlm.nih.gov/SNP). The −159 (rs2569190; also reported as CD14-260) in the promoter region of CD14 is the polymorphism most often described. From genetic perspective, the substitution of C-159T results in elevated transcriptional activity and accordingly high serum CD14 levels [10]. Therefore, candidacy of CD14 for sepsis is well-suggested and its C-159T polymorphism has been reported to be associated with sepsis by some studies [10]–[12]. Generally, studies with insufficient sample sizes and limited power may account for such inconsistency [2]. We set to analyze published data and hope to provide more power and precise estimation of the clinical impact of CD14-159C/T polymorphism, to determine whether CD14-159C/T is associated with the risk of sepsis or sepsis-related mortality by a meta-analysis.

## Materials and Methods

This meta-analysis was performed according to the recommendations of the Preferred Reporting Items for Systematic Reviews and Meta-analysis criteria [13]. We used the term “sepsis” to encompass clinical conditions such as sepsis, severe sepsis, septic shock, and septicemia.

### Search strategy

The PubMed, Medline, Embase, Web of Knowledge and HuGE Navigator databases were searched in order to identify all published case-control studies up to June 15 2012 that had evaluated the associations between CD14 polymorphism and sepsis. The Medical Subject Headings and key words used for search were “CD14 or cluster of differentiation-14” and “sepsis or severe sepsis or septic shock or septicemia” and “polymorphism or variant or mutation”. We evaluated all associated publications to retrieve the most eligible literature. The references were searched manually to identify additional eligible studies. Authors were contacted directly regarding crucial data not reported in original articles. Unpublished reports and articles written in non-English languages were excluded.

### Study selection

The inclusion criteria were: (1) independent case-control design for human; (2) evaluate the association between CD14-159C/T and the risk or mortality of sepsis; (3) the number or frequency of genotypes was given in detail or obtained by contacting the authors.

The exclusion criteria were: (1) studies with insufficient information were excluded, for example, genotype frequency or number not reported; (2) abstract, comment, review and editorial; (3) duplicate publications, only the most recent or complete study was included after careful examination. When a study reported the results on different ethnicities, we treated them as separated studies.

Two researchers (AQ Zhang and CL Yue) evaluated the titles and abstracts of identified publications. Potentially relevant publications were retrieved and further evaluated. The authors and outcomes of the studies were not blinded to the researchers. Final inclusion of studies in the meta-analysis was determined by agreement of both researchers. Agreement between researchers was evaluated by using a κ statistic. Strength of agreement as evaluated by the κ statistic was defined as slight (0.00–0.20), fair (0.21–0.40), moderate (0.41–0.60), substantial (0.61–0.80), or almost perfect (0.81–1.00) [14].

### Data extraction and methodological approach

To minimize the bias and improve the reliability, two researchers extracted data with the above inclusion and exclusion criteria independently and reached a consensus. Information such as first author's name, publication year, country origin and ethnicity of study population, genotyping method, sepsis type, genotype number or allele frequency for case and control, adjusting factors for statistical analysis were collected from each study using a standardized data collection protocol.

### Assessment of study quality

The quality of the studies was independently assessed by two researchers according to a set of predetermined criteria which was extracted and modified from previous studies [15] (Table S1). These scores were based on traditional epidemiological considerations, as well as complex disease genetic issues [16]. Any disagreement was resolved by discussion between the two researchers. Scores ranged from the lowest zero to the highest 8. Articles scoring <6 were classified as “low quality”, and those ≥ 6 as “high quality”. To assess methodological quality, included articles were examined for: 1) inclusion of a statement about Hardy-Weinberg equilibrium; 2) description of the primer sequence or reference to a previous publication, which included the sequence; 3) blinding to genotype and clinical status; and 4) provision of a definition of sepsis according to the American College of Chest Physicians/Society of Critical Care Medicine guidelines [17].

### Statistical analysis

We first assessed Hardy-Weinberg equilibrium (HWE) for each study using Chi-square test in control group. The odds ratio (OR) and its 95% confidence interval (95%CI) were used to assess the strength of the association between CD14-159C/T and sepsis susceptibility or mortality based on genotype frequencies in cases and controls. The pooled ORs were performed for dominant (CT+TT versus CC), recessive (TT versus CT+CC), and allelic (T versus C) genetic model, respectively. The significance of pooled ORs was tested by Z-test (p<0.05 was considered statistically significant). Heterogeneity between studies was assessed using I^2^ value and χ^2^-based Q-test [18], where I^2^ values approaching zero (0%) indicated no observed heterogeneity and larger values increasing heterogeneity. p≥0.10 for the Q-test indicated a lack of heterogeneity across studies, allowing to use the fixed-effects model (the Mantel-Haenszel method) [19]; otherwise, the random-effects model was used (the DerSimonian and Laird method) [20]. The sources of heterogeneity were investigated using subgroup analyses carried out by ethnicity (Asian vs. European populations) and sepsis type (sepsis, severe sepsis, and septic shock). Sensitivity analysis was performed to assess the stability of the results, namely, a single study in the meta-analysis was deleted each time to reflect the influence of the individual data set to the pooled OR. Moreover, sensitivity analysis was also performed, excluding studies whose allele frequencies in controls exhibited significant deviation from the HWE, given that the deviation may denote bias. Potential publication bias was estimated using visual inspection of the funnel plot [21], and Egger's linear regression test [22]. All p values were two-sided, and all statistical analyses were performed using Review Manager 5.0 (Cochrane Collaboration, http://www.cc-ims.net/RevMan/relnotes.htm/) and STATA11.0 software (StataCorp LP, College Station, Texas, USA).

To ensure reliability and accuracy of the results, two researchers entered the data into the software program independently and reached a consensus.

## Results

### Characteristics of eligible studies

**Figure 1:**
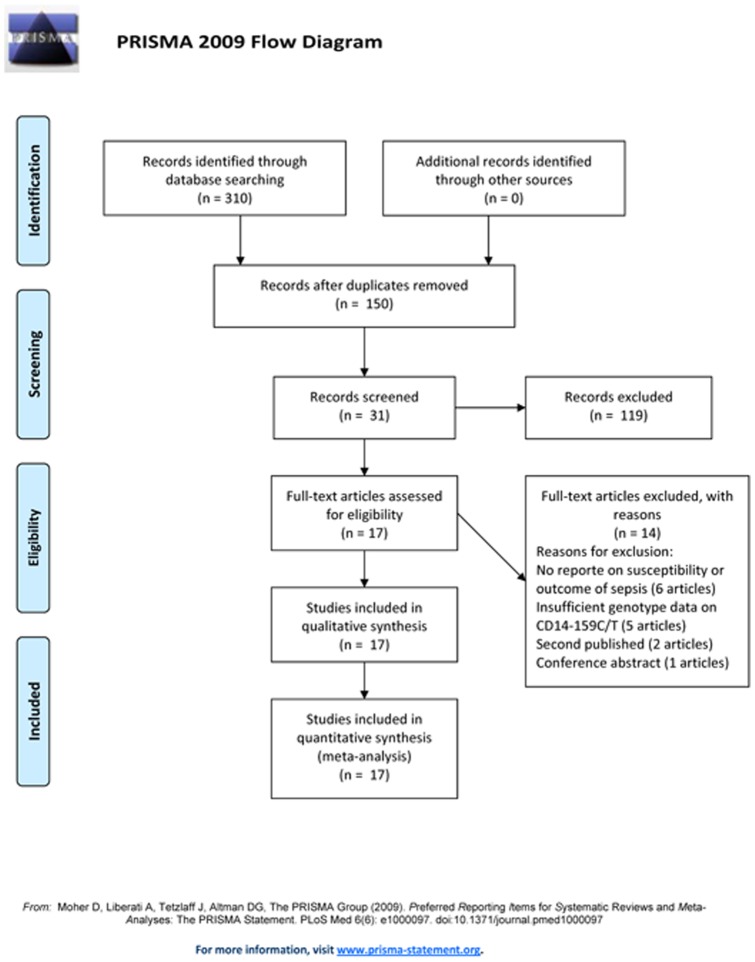
Flow diagram of study identification.

A total of 310 articles were found between January 2000 and June 2012 (57 from PubMed, 51 from Medline, 63 from Embase, 106 from Web of Science, and 31 from HuGE Navigator database). After removing 160 duplications and reading the abstracts, 31 articles were remained for which the full-text article was retrieved. Of the 31 articles, 14 were excluded, of which 6 articles did not report on susceptibility or outcome of sepsis [11], [23]–[27], 5 articles did not supplied detailed genotype data or genotype frequency information by contacting the authors [12], [28]–[31], 2 articles due to second published [32], [33] and 1 conference abstract [34]. Finally, 17 relevant articles were included in final meta-analysis (Flow diagram shown in Figure 1). The researchers had substantial agreement on articles for inclusion with a κ statistic of 0.85 (95%CI: 0.70–0.95). The study was judged to be of good quality if the total score was over 6, otherwise, of poor quality. The total score of all studies was over 6 (Table S2).

Information of the 17 included articles including the first author, publication year, country and ethnicity, sepsis type, sample size (case/control), genotype data, and quality score was listed in Table 1. Among the 17 publications, 12 studies were performed in European populations, five in Asian populations. The majority of studies focused on adult, whereas two studies were performed in the pediatric population [35], [36]. Sepsis was defined sepsis (11 studies), severe sepsis (4 studies), and septic shock (2 studies). 16 studies evaluated susceptibility to sepsis, 1 evaluated sepsis mortality, and 3 studies evaluated both.

**Table 1:** Characteristics of the studies included in the meta-analysis.

Study	Country	Ethnicity	Sepsis type	Sources of	Cases/		Cases		Controls		Quality	
				controls	Controls	CC	CT	TT	CC	CT	TT	score
Hubacek 2000	Germany	European	severe sepsis	healthy	204/247	53	111	40	64	128	55	7
Hubacek 2000	Germany	European	non-survivors	survivors	97/107	21	59	17	32	52	23	7
Gibot 2002	France	European	septic shock	healthy	90/122	19	43	28	44	52	26	7
Gibot 2002	France	European	non-survivors	survivors	50/40	5	25	20	14	18	8	7
Heesen 2002	Netherlands	European	severe sepsis	trauma	14/44	5	5	4	15	22	7	7
Ahrens 2004	Germany	European	sepsis	VLBW	50/306		36^a^	14		248 ^a^	58	7
Barber 2004	American	European	severe sepsis	burn	36/123	16	17	3	36	60	27	8
Nakada 2005	Japan	Asian	sepsis	critically ill	86/111	15	43	28	27	42	42	7
Zhang 2005	China	Asian	septic shock	ASP	33/76	19	9	5	47	20	9	7
D'Avila 2006	Brazil	European	sepsis	critically ill	52/33	17	19	16	10	16	7	8
Jessen 2007	Denmark	European	non-survivors	sepsis	62/252	18	33	11	74	123	55	8
de Aguiar 2008	Brazil	European	sepsis	healthy	14/30	3	7	4	7	19	4	7
Gu 2008	China	Asian	sepsis	trauma	42/63	5	23	14	16	35	12	8
Yuan 2008	Australia	European	sepsis	healthy children	85/409	30	35	20	100	201	108	6
Fallavena 2009	Brazil	European	sepsis	critically ill	343/171	108	172	63	49	78	44	8
Lin 2009	China	Asian	severe sepsis	burn	22/42	4	9	9	8	23	11	8
Shalhub 2009	American	European	sepsis	SIRS patients	147/451		48%^b^			44% ^b^		8
Davis 2010	American	European	sepsis	healthy	28/53	7	13	8	13	30	10	6
Shimada 2011	Japan	Asian	sepsis	ICU patients	123/101	29	57	37	27	43	31	8
Shimada 2011	Japan	Asian	non-survivors	survivors	21/102	5	5	11	24	52	26	8

ASP: acute severe pancreatitis, VLBW: very low birth weight, SIRS: systemic inflammatory response syndrome, ICU: Intensive Care Unit, ^a^ represents the number of CC+CT genotype, ^b^ represents the frequency of the T allele.

Study quality characteristics are shown in Table 2. Whereas the majority of the studies provided detailed information about the primer (16 of 17 [94%]) and used a sepsis definition consistent with the American College of Chest Physicians/Society of Critical Care Medicine guidelines (14 of 17 [82%]), only the minority of the studies were reported to be “blinded” (7 of 17 [41%]). The results of Hardy-Weinberg equilibrium test for the distribution of the genotype in control population were in agreement in 15 of 17 studies.

**Table 2:** Methodologic quality.

Study	Year	HWE^a^	Primer^b^	Blinding^c^	Sepsis Definition^d^
Hubacek JA	2000	+	+	−	+
Heesen M	2002	+	+	−	+
Gibot S	2002	+	+	−	+
Barber RC	2004	+	+	−	+
Ahrens P	2004	+	+	−	−
Nakada TA	2005	−	+	−	+
Zhang DL	2005	−	+	−	+
D'Avila LC	2006	+	+	+	+
Jessen KM	2007	+	+	+	+
de Aguiar BB	2008	+	+	+	+
Gu W	2008	+	+	+	+
Yuan FF	2008	+	+	−	−
Fallavena PR	2009	+	+	+	+
Lin J	2009	+	+	−	+
Shalhub S	2009	+	+	+	+
Davis SM	2010	+	−	−	−
Shimada T	2011	+	+	+	+

*a* Studies assessed if their cohort and control groups were in Hardy-Weinberg equilibrium (HWE); *b* authors published the primer sequence(s) or provided a sufficient reference; *c* studies performed genotyping whilst blind to the clinical status of the patient; *d* studies used sepsis (-subtype) definition according to the American College of Chest Physicians/Society of Critical Care Medicine guidelines [17].

Different genotyping methods were used in these studies, including the classical polymerase chain reaction-restriction fragment length polymorphism (PCR-RFLP) in 11 of 17 studies, specific fluorescence-labeled probes in 4 studies, pyrosequencing in 1 study and microsphere based assay in 1 study.

### Association between CD14-159C/T and sepsis

Sixteen studies with 1369 cases and 2382 controls determined the association between −159C/T polymorphism and sepsis risk [10], [35]–[50]. The overall estimated ORs for all studies combined were 1.01, 1.10, and 1.05 for dominant, recessive, and allelic genetic model respectively (Table 3), these associations were not statistically significant (P>0.10). Next, we performed subgroup analysis according to ethnicity. Similar results were obtained in European populations, but a borderline significant effect for Asian populations was found under the dominant genetic model (OR = 1.38, 95%CI = 0.96–1.98, P = 0.08). Further, stratification by subtypes of sepsis indicated that the statistically significant association was only found among septic shock patients (Dominant model: OR = 1.72, 95%CI = 1.05–2.83, P = 0.03; Allelic model: OR = 1.52, 95%CI = 1.09–2.12, P = 0.01). Considering to the relatively small number of studies available for septic shock (n = 2), a random-effects model was also used to calculate the pooled ORs and the results remained to be significant (Dominant model: OR = 1.70 95%CI = 0.99–2.92, P = 0.05, Allelic model: OR = 1.52 95%CI = 1.09–2.12 P = 0.01).

**Table 3:** Meta-analysis of CD14-159C/T with sepsis susceptibility.

Comparisons		Sample	Size	No. of studies		Test of association				Test of heterogeneity	
		Case	Control		OR (95%CI)	Z	P-value	Model*	?2	P-value	I^2^(%)
Risk of sepsis											
Overall	CT+TT vs. CC	1172	1625	14	1.01(0.85–1.21)	0.15	0.88	F	17.63	0.17	26%
	TT vs. CT+CC	1222	1931	15	1.10(0.87–1.41)	0.80	0.42	R	21.65	0.09	35%
	T vs. C	2638	4152	15	1.05(0.91–1.22)	0.69	0.49	R	23.70	0.05	41%
Asian	CT+TT vs. CC	306	393	5	**1.38(0.96–1.98)**	**1.74**	**0.08**	F	1.76	0.78	0%
	TT vs. CT+CC	306	393	5	1.09(0.78–1.51)	0.51	0.61	F	4.48	0.34	11%
	T vs. C	612	786	5	1.17(0.94–1.45)	1.40	0.16	F	2.89	0.58	0%
European	CT+TT vs. CC	866	894	9	0.91(0.74–1.13)	0.84	0.40	F	12.14	0.15	34%
	TT vs. CT+CC	916	1538	10	1.09(0.79–1.51)	0.51	0.61	R	16.78	0.05	46%
	T vs. C	2026	3366	10	1.00(0.83–1.21)	0.01	0.99	R	18.82	0.03	52%
Sepsis	CT+TT vs. CC	650	870	8	0.96(0.76–1.21)	0.33	0.74	F	8.93	0.26	22%
	TT vs. CT+CC	700	1176	9	0.99(0.79–1.24)	0.08	0.94	F	12.81	0.12	38%
	T vs. C	1840	2844	9	1.01(0.89–1.14)	0.09	0.93	F	11.46	0.18	30%
Severe sepsis	CT+TT vs. CC	276	456	4	0.88(0.62–1.23)	0.76	0.45	F	2.27	0.52	0%
	TT vs. CT+CC	276	456	4	0.89(0.61–5.82)	0.63	0.53	F	5.96	0.11	50%
	T vs. C	552	912	4	0.91(0.73–1.13)	0.85	0.40	F	5.12	0.16	41%
Septic shock	CT+TT vs. CC	123	198	2	**1.72(1.05–2.83)**	**2.16**	**0.03**	F	1.15	0.29	13%
	TT vs. CT+CC	123	198	2	1.44(0.86–2.41)	1.40	0.16	F	0.67	0.42	0%
	T vs. C	246	396	2	**1.52(1.09–2.12)**	**2.46**	**0.01**	F	0.63	0.43	0%

Risk of mortality											
Overall	CT+TT vs. CC	230	501	4	**1.44(0.98–2.11)**	**1.86**	**0.06**	F	6.18	0.10	51%
	TT vs. CT+CC	230	501	4	1.41(0.68–2.94)	0.92	0.36	R	9.60	0.02	69%
	T vs. C	460	1002	4	1.36(0.89–2.08)	1.44	0.15	R	8.65	0.03	65%

* : F represents fixed-effects model (the Mantel-Haenszel method), R represents random-effects model (the DerSimonian and Laird method). p≥0.10 for the Q-test indicated a lack of heterogeneity across studies, allowing to use the fixed-effects model; otherwise, the random-effects model was used.

Regarding sepsis-related mortality risk, only four studies containing 731 sepsis patients (230 non-survivors and 501 survivors) contributed to our meta-analysis. Table 3 indicates that the odds of sepsis mortality are increased by 40% in all three models, but only dominant genetic effect approached statistical significance (OR = 1.44, 95%CI = 0.98–2.11, P = 0.06). In addition, the upper end of the 95%CI of OR is up to 2.94 under the recessive genetic model (OR = 1.41, 95%CI = 0.68–2.94, P = 0.36), also indicating the possibility of almost a three-fold increased mortality risk. Due to the small number of studies to sepsis mortality, further subgroup analysis by ethnicity and sepsis type was not done.

### Heterogeneity analysis

For sepsis risk, there was a slight between-study heterogeneity in the recessive and allelic genetic model, respectively (Recessive model: I^2^ = 35%, P = 0.09; Allelic model: I^2^ = 41%, P = 0.05) (Table 3). When the studies by Fallavena et al. [41] and Barber et al. [47] were excluded, the heterogeneity effectively decreased (Recessive model: I^2^ = 5%, P = 0.40; Allelic model: I^2^ = 15%, P = 0.29), whereas the pooled results were not materially changed. For sepsis-related mortality risk, there was also statistically significant heterogeneity under the recessive and allelic genetic model (Recessive model: I^2^ = 68.7%, P = 0.02; Allelic model: I^2^ = 65.3%, P = 0.03), which did not further analyzed due to the small number of studies (n = 4).

### Sensitivity Analysis

A single study involved in the meta-analysis was removed each time to reflect the influence of its individual data set on the pooled ORs, and the corresponding pooled ORs were not materially altered. For the comparisons between CD14-159 and sepsis risk, the exclusion of the two studies by Zhang et al. [45] and Nakada et al. [46], whose genotypic distribution in controls deviated from HWE, did not change the results significantly. Sensitivity analysis suggested the robustness of our results.

### Publication bias analysis

**Figure 2:**
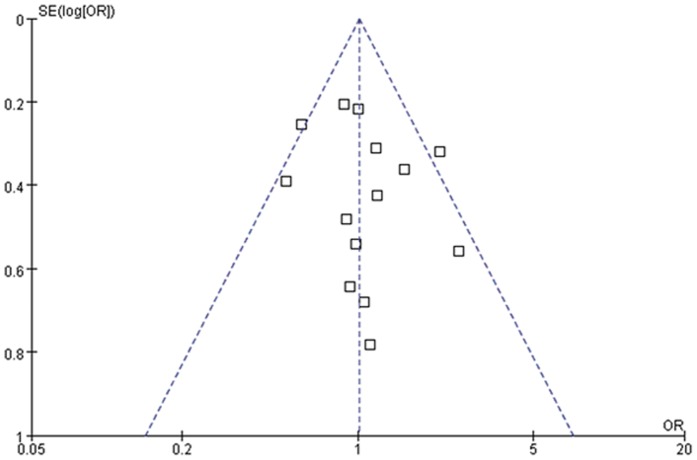
Forest plot of sepsis susceptibility associated with CD14-159C/T under the dominant genetic model. The horizontal and vertical axis correspond to the OR and confidence limits. OR: odds ratio; SE: standard error.

**Figure 3:**
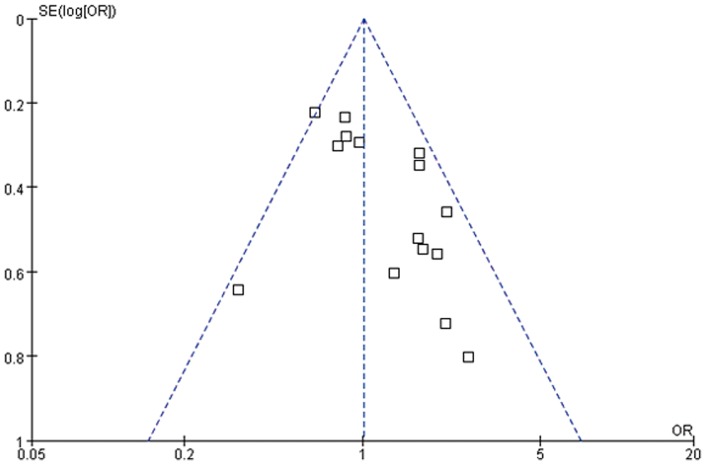
Forest plot of sepsis susceptibility associated with CD14-159C/T under the recessive genetic model. The horizontal and vertical axis correspond to the OR and confidence limits. OR: odds ratio; SE: standard error.

**Figure 4:**
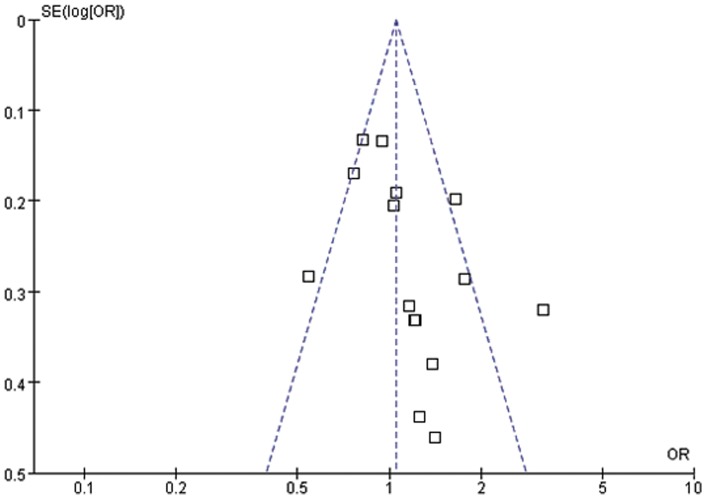
Forest plot of sepsis susceptibility associated with CD14-159C/T under the allelic genetic model. The horizontal and vertical axis correspond to the OR and confidence limits. OR: odds ratio; SE: standard error.

Publication bias was assessed by performing funnel plot qualitatively and Egger's test quantitatively. The shape of the funnel plots seemed slightly asymmetrical for sepsis risk in overall meta-analysis (Figure 2, 3 and 4). Egger's test did not show obvious evidence of publication bias for sepsis risk under dominant and allelic genetic model (P = 0.39 and P = 0.22). However, slight publication bias was found under recessive model (P = 0.05). Further, Funnel plot and Egger's test were not applied in sepsis mortality due to the small number of studies (4 studies).

## Discussion

Previous studies have reported the CD14-159C/T to be associated with sepsis with inconsistent results. In the present study, we identified 17 genetic association studies and used meta-analysis to evaluate the association of CD14-159C/T polymorphism with sepsis under the dominant, recessive and allelic genetic model respectively. The pooled ORs and 95%CIs under different comparison models did not show strongly significant association of CD14-159C/T polymorphism with sepsis risk in overall populations.

Different CD14-159T allele frequencies have been reported in different ethnic populations from the International HapMap Project, which were 0.474, 0.500, 0.487, and 0.293 among Utah residents with Northern and Western European ancestry, Han Chinese in Beijing, China, Japanese in Tokyo, Japan, and Yoruba in Ibadan, Nigeria, respectively. We performed a stratified analysis by ethnicity to determine whether the association between T allele and sepsis differed by population. We noted that the effect of CD14-159C/T on sepsis risk was more pronounced in Asians under the dominant genetic model (OR = 1.38, 95%CI = 0.96–1.98, P = 0.08), although there was only borderline significant difference. However, a final conclusion cannot be drawn because the number from Asian studies analyzed for sepsis risk is very small (n = 5). Further prospective well designed and larger sample size studies to address this question are warranted based on this meta-analysis.

In the subgroup analysis, we also examined that whether the effect of variant T allele differed depending on the way in which sepsis was described (sepsis, severe sepsis, or septic shock). We found that the way in which sepsis was described affected the association between CD14-159C/T and susceptibility to sepsis. The significant association was in septic shock patients under the dominant and allele model (OR = 1.72, 95%CI = 1.05–2.83, P = 0.03; OR = 1.52, 95%CI = 1.09–2.12, P = 0.01, respectively), which was not altered when the random-effects model was used. In addition, the upper end of the 95%CI of OR is up to 5.82 among severe sepsis patients under the recessive genetic model (OR = 0.89, 95%CI = 0.61–5.82, P = 0.63), indicating the possibility of almost a six-fold increased sepsis risk, which would almost certainly be clinically significant. These results indicate the possibility of a clinically important relationship that needs to be explored more fully in a larger sample size and more powerful study.

For sepsis-related mortality risk, although the effect size estimates are not statistically significant at the conventional arbitrary alpha = 0.05 level, the odds of sepsis mortality are increased by 40% in all three models, and the dominant genetic model approached statistical significance (OR = 1.44, 95%CI = 0.98–2.11, P = 0.06). Since there were only four studies performed in sepsis-related mortality risk, subgroup analyses could not be conducted and more studies should be designed to analyze these conditions.

There were modest heterogeneities in the overall comparisons for CD14-159C/T polymorphism and sepsis risk. To explore the source of heterogeneities, we found that all I^2^ values were decreased after excluding two studies by Fallavena et al. [41] and Barber et al. [47]. The results suggested that the two studies might be the major source of the heterogeneities. However, heterogeneity did not seem to influence the results, because the significance of the result was not altered after excluding the two studies. In addition, the heterogeneities were also obvious decreased though subgroup analysis by sepsis subtype, which indicated the severity of sepsis might influence the heterogeneities. For sepsis-related mortality risk, there were notable heterogeneities under any genetic model (I^2^>50%, P≤0.10), which may be caused by the different ethnicities and sepsis subtypes of the four studies. Moreover, we carried out sensitivity analyses. Removal of each study or the studies deviating from HWE did not alter the associations with sepsis risk and mortality risk, suggesting the reliability of these results.

One of the important concerns in meta-analysis is publication bias. Because meta-analysis reviews quantitative data from numerous studies, the publication bias effect of the literature incorporated in the study can bias the meta-analytic outcome. Although the Egger's test did not show significant publication bias for sepsis risk, we found the shape of the funnel plot was slightly asymmetrical. Thus, the results should be interpreted cautiously and more studies are still needed to confirm the finding from this meta-analysis.

There are several limitations in the present study. First, the number of included studies was small. Some unpublished reports, non-English articles, and studies without sufficient information were not included in our meta-analysis, which may bias our results. Second, although stratified analyses are an important approach to attempt to explain heterogeneity in an effect, there were a small number of studies in each stratum, thus limiting the interpretation of these analyses. Third, sepsis is a complex clinical syndrome resulting from a systemic inflammatory response to bacteria and/or bacterial products. Several factors such as age, sex, category of pathogens and control source are closely related to the susceptibility or progression of sepsis. Meta-regression would have been an optimal approach to better examine potential confounders. However, these factors cannot be evaluated in the meta-analysis due to the limited information. Fourth, given the number of DNA banks and ease of examining a large number of polymorphisms in a genetic association study, there is a possibility that such negative analyses of CD14-159C/T and sepsis have not been published. Finally, many genes were associated with sepsis [2], [51]. We could not address gene-gene interactions in this meta-analysis due to the lack of the related information.

Nonetheless, to the best of our knowledge, the present study represents the first meta-analysis investigating the relationship between CD14 gene C-159T polymorphism and risk of sepsis. We suggest that such an approach of combining the results of association studies may help us to better understand the effect of polymorphisms on disease outcomes.

### Conclusion

The results of this meta-analysis suggest that the CD14-159C/T polymorphism may not significantly influence the risk for sepsis in overall populations. However, a slight association was found in Asian populations and septic shock patients despite a limited number of studies included in the analysis. Larger sample size and well-designed genetic association studies are warranted to confirm these results.

## Supporting Information

Table S1 **The criteria of quality evaluation for included studies.**
(DOC)

Table S2 **The results of quality evaluation for included studies.**
(XLS)
