# Supplementary material for: Association between CD14 Promoter -159C/T Polymorphism and the Risk of Sepsis and Mortality: A Systematic Review and Meta-Analysis
Source: PLoS One. 2013 Aug 19;8(8):e71237. doi: 10.1371/journal.pone.0071237 (PMC3747171; doi:10.1371/journal.pone.0071237)
Supplement: Table S1 — The criteria of quality evaluation for included studies. (DOC) [file pone.0071237.s001.doc]

**Table S1. The criteria of quality evaluation for included studies.**

1. Is the Case Definition Adequate?
2. Yes, with independent validation (1 score)
3. Yes, but record linkage or self-report with no reference to primary record (0 score)
4. No description (0 score)
5. Representativeness of the Cases

a) Consecutive or obviously representative series of cases (1 score)

b) Potential for selection biases or not stated (0 score)

1. Selection of Controls
2. Community controls or hospital-based controls (1score)
3. Hospital controls, but derived from a hospitalized population (1 score).
4. No description (0 score)
5. Definition of Controls

a) No history of disease (1score)

b) No description of source (0 score)

1. Comparability of Cases and Controls on the Basis of the Design or Analysis

a) Study controls for the main confounding factors (1 score)

b) Study controls for any confounding factors (1 score)

c) No control (0 score)

1. Ascertainment of Exposure

a) Definite records (such as surgical records) or structured interview where blind to case/control status (1 score)

b) Written self report or medical record only (0 score)

1. No description (0 score)
2. Non-Response Rate
3. Consistency for cases and control (1 score)
4. Description for non respondent (0 score)
5. Inconsistency or No description (0 score)
6. Method of determination for cases and controls
7. Consistency (1 score)
8. Inconsistency (0 score)

Note:

If the total scores are over 6, the quality of the paper is good, otherwise, the quality is poor (score: 0-5).
